# The Association of Cytokines IL-2, IL-6, TNF-α, IFN-γ, and IL-10 With the Disease Severity of COVID-19: A Study From Bangladesh

**DOI:** 10.7759/cureus.57610

**Published:** 2024-04-04

**Authors:** Farzana Islam, Shahriar Habib, Khaja Badruddza, Mahatabur Rahman, Mohammad R Islam, Sharmin Sultana, Afzalun Nessa

**Affiliations:** 1 Division of Laboratory Service, Sheikh Hasina National Institute of Burn and Plastic Surgery, Dhaka, BGD; 2 Department of Microbiology, Sher-E-Bangla Medical College, Barishal, BGD; 3 Department of Biochemistry, Sher-E-Bangla Medical College, Barishal, BGD; 4 Department of Gastroenterology, Sheikh Russel National Gastro-Liver Institute, Dhaka, BGD; 5 Department of Anesthesia, Mainamoti Medical College and Hospital, Cumilla, BGD; 6 Department of Virology, Bangabandhu Sheikh Mujib Medical University, Dhaka, BGD; 7 Department of Virology, Bangabandhu Sheikh Mujjib Medical University, Dhaka, BGD

**Keywords:** il-10, ifn-γ, tnf-α, il-6, il-2, sars-cov-2

## Abstract

Introduction

Clinically, the early prediction of the severity of COVID-19 is often challenging, as a dramatic change in severity can occur without warning. The severity of COVID-19 disease is associated with an increased level of inflammatory mediators, including cytokines. This study aimed to evaluate the association of the levels of cytokines interleukin (IL)-2, IL-6, tumor necrosis factor-alpha (TNF-α), interferon-gamma (IFN-γ), and IL-10 with the severity of COVID-19 in Bangladesh.

Materials and methods

This cross-sectional study included a total of 60 confirmed cases of COVID-19, comprising 30 severe cases (Group A) and 30 non-severe cases (Group B), and 10 healthy individuals (Group C) attending Bangabandhu Sheikh Mujib Medical University (BSMMU) from March 2021 to February 2022. The cytokine assay was performed using the human Th1/Th2/Th17 cytokine kit BD cytometric bead array (CBA) on the BD Accuri C6 Plus flow cytometer. Statistical analysis was conducted using IBM SPSS Statistics for Windows, Version 23 (Released 2015; IBM Corp., Armonk, New York).

Results

The mean ages of the patients in Groups A, B, and C were 60.73±5.97, 57.13±7.68, and 48.10±9.13 years, respectively, with a male predominance in all groups. The mean IL-2, IL-6, IL-10, and TNF-α levels had a positive correlation with the increased age group and male gender, although it was not statistically significant. The mean IL-6 and IFN-γ levels were significantly higher among severe cases (216.95±147.78 and 0.98±0.95 pg/mL, respectively) compared to non-severe cases (94.29±128.79 and 0.41±0.61 pg/mL, respectively) and healthy individuals (1.08±1.97 and 0.15±0.28 pg/mL, respectively). Furthermore, the anti-inflammatory cytokine IL-10 was also significantly higher among severe cases (17.92±21.87 pg/mL) compared to non-severe cases (5.38±6.73 pg/mL) and healthy individuals (1.62±1.65 pg/mL).

Conclusion

IL-6, IFN-γ, and IL-10 have a significant association with the severity of COVID-19 disease. Clinicians treating patients with COVID-19 can consider the level of these cytokines as biomarkers of severity.

## Introduction

Severe acute respiratory syndrome coronavirus 2, or SARS-CoV-2, was first identified in Wuhan, China, in 2019, and coronavirus disease 2019 (COVID-19), caused by SARS-CoV-2, was declared a public health emergency of international concern initially and a pandemic later on by the World Health Organization (WHO) [1]. COVID-19 causes a hyper-inflammatory condition responsible for various tissue damages and critical conditions like acute respiratory distress syndrome (ARDS) and multiorgan failure [2].

Cytokines are proteins or signaling peptides that play various potent biological functions at picomolar concentrations [3]. Pro-inflammatory cytokines such as interleukin (IL)-2, IL-6, tumor necrosis factor-alpha (TNF-α), and interferon-gamma (IFN-γ) are important for initiating a response to infection, but anti-inflammatory cytokines such as IL-10 are released during any sustained infection to control inflammation and maintain immune homeostasis [4]. Activated monocytes and macrophages are the primary sources of cytokines, and the virulence of the process is related to cytokine levels [5].

COVID-19 patients with severe disease tend to have increased levels of cytokines, which may result in a cytokine storm [6]. Infected epithelial cells or immune cells by SARS-CoV-2 release pro-inflammatory cytokines; these cytokines recruit innate immune cells and activate adaptive immune cells, further inducing myelopoiesis and granulopoiesis that intensify lung and epithelial damage [7].

Pro-inflammatory cytokines such as interleukins IL-2, IL-6, tumor necrosis factor-alpha (TNF-α), interferon-gamma (IFN-γ), and many other molecules, including chemokines, play a significant role in the pathophysiology of COVID-19 [8]. Excessive IL-6 levels, acting as an axis for cytokine storms in COVID-19 patients, correlate with severe diseases [9]. On the contrary, IL-10 has the ability to induce innate and adaptive immune responses, so it is widely known to be anti-inflammatory and restrains pro-inflammatory responses to avert tissue damage [10].

In summary, pro-inflammatory cytokines play a significant role in the pathophysiology of COVID-19, and severe COVID-19 patients have an increased tendency toward the production of cytokines, leading to a cytokine storm. Cytokines such as IL-2, IL-6, TNF-α, IFN-γ, and IL-10 could serve as predictors for early prediction of the severity of COVID-19; hence, this study aimed to evaluate the association of cytokine levels IL-2, IL-6, TNF-α, IFN-γ, and IL-10 with the severity of COVID-19 in Bangladesh.

## Materials and methods

This cross-sectional study was conducted from March 2021 to February 2022. The study was reviewed and approved by the Institutional Review Board (IRB) of Bangabandhu Sheikh Mujib Medical University (reference: BSMMU/2021/6587, registration number: 3514, date: July 24, 2021). Participants were selected during the COVID-19 pandemic in 2021, specifically from May to September. A total of 60 patients who attended the COVID Intensive Care Unit (ICU) and COVID Unit (Cabin Block) of Bangabandhu Sheikh Mujib Medical University (BSMMU) were included in the study. RT-PCR-positive severe and non-severe COVID-19 adult patients (18 years of age or older) of both genders with symptoms as per the WHO Guideline, 2021, were included in this study within five to ten days of a positive COVID-19 RT-PCR test result. Critical COVID-19 patients, patients with a known case of tuberculosis, patients with a known case of chronic diseases (such as rheumatological diseases, chronic kidney disease, connective tissue disease, vasculitis, or a diagnosed case of malignancy), and pregnant women were excluded from the study.

The selected patients were distributed into two groups, Group A and Group B, according to the WHO COVID-19 Clinical Management Guidelines 2021. Group A consisted of 30 patients with severe COVID-19 symptoms, and Group B consisted of 30 patients with non-severe COVID-19 symptoms. Furthermore, a total of 10 apparently healthy participants without any history of COVID-19 in the last six months were included as controls in Group C. Participants were thoroughly informed of the objectives and detailed procedures of the study before giving their written consent. Data were collected using a pre-prepared data collection sheet, and approval was obtained from the Institutional Review Board (IRB) of Bangabandhu Sheikh Mujib Medical University (BSMMU).

Approximately 3 mL of venous blood was collected from each participant, maintaining all aseptic precautions and labeling it properly. Serum was first separated by centrifugation at 1400 rpm for five minutes, then collected in microcentrifuge tubes and labeled, and lastly preserved at -70°C until further processing. Later on, from the stored serum samples, the concentration of selected cytokines (IL-2, IL-6, IL-10, TNF-α, and IFN-γ) was estimated using the human Th1/Th2/Th17 cytokine kit by the CBA method in the Accuri C6 BD plus flow cytometer (BD Biosciences, Franklin Lakes, New Jersey) following the manufacturer's instructions. Flow cytometric data analysis was performed using FCAP Array software, version 3.0 (BD Biosciences).

Statistical analysis was performed using IBM SPSS Statistics for Windows, Version 23 (Released 2015; IBM Corp., Armonk, New York), and a p-value of less than 0.05 was considered significant. Cytokine comparisons between groups were analyzed by unpaired t-tests. Comparisons between severe and non-severe infections in both Group A and Group B were analyzed using an independent t-test.

## Results

The demographic data of the study participants are presented in Table 1. The mean age of the participants in Group A was 60.73±5.97 (SD) years, in Group B was 57.13±7.68 (SD) years, and in Group C was 48.10±9.13 (SD) years. Among the three groups, 73.3% (n = 22), 50.0% (n = 15), and 60.0% (n = 6) were male, respectively. Most of the study participants were from urban areas during the COVID-19 episodes, with 56.7% in Group A and 53.3% in Group B. Participants were classified into low, medium, and high socioeconomic statuses according to their monthly income. Most of the study participants belonged to middle-income families, with 56.7% in Group A and 66.7% in Group B.

**Table 1:** Sociodemographic characteristics of the study participants (n=70)

Variables	Group A (N=30), n (%)	Group B (N=30), n (%)	Group C (N=10), n (%)
Gender			
Male	22 (73.3)	15 (50.0)	6 (60.0)
Female	8 (26.7)	15 (50.0)	4 (40.0)
Residence			
Urban	17 (56.7)	16 (53.3)	7 (70.0)
Rural	13 (43.3)	14 (46.7)	3 (30.0)
Socioeconomic status			
Low	0 (0.0)	0 (0.0)	2 (20.0)
Middle	17 (56.7)	20 (66.7)	6 (60.0)
High	13 (43.3)	10 (33.3)	2 (20.0)

Regarding presenting complaints, 100% of patients in both groups had a fever. Cough was present in 96.7% (n = 29) of patients with severe COVID-19 (Group A) and 90.0% (n = 27) of patients with non-severe COVID-19. 3.3% (n = 1) of the patients in Group B had anosmia as a complaint, while none of the patients in Group A had this complaint. The complaints of respiratory distress, sore throat, and runny nose were found to be 93.33%, 0%, and 0%, respectively, in Group A, and 0%, 73.3%, and 56.7% among Group B patients. The presence of respiratory distress, sore throat, and runny nose was higher in Group A patients, which was found to be statistically highly significant.

**Figure 1:**
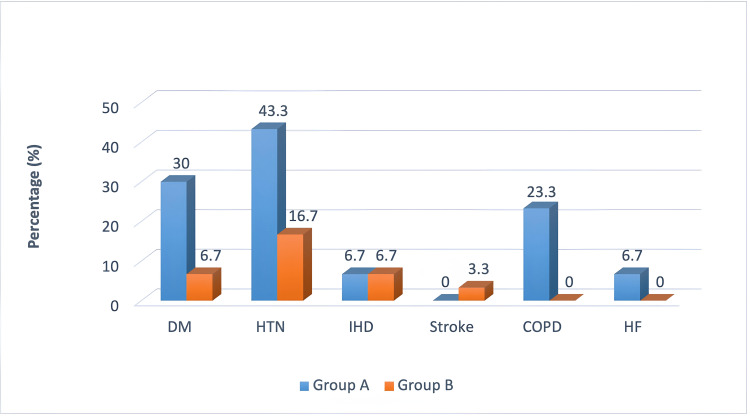
Presence of co-morbidities among Group A (n=30) and Group B patients (n=30) (p=0.001) DM: diabetes mellitus; HTN: hypertension; IHD: ischemic heart disease; COPD: chronic obstructive pulmonary disease; HF: heart failure.

Figure 1 shows comorbidities among different groups. All participants in Group A had at least one comorbidity. Among the participants in Group A, 30%, 43.3%, 23.3%, and 6.7% had the comorbidity of diabetes mellitus (DM), hypertension (HTN), chronic obstructive pulmonary disease (COPD), and heart failure (HF), respectively, while among the participants in Group B, it was 6.7%, 16.7%, 0%, and 0%, respectively. However, the percentage of ischemic heart disease (IHD) (6.7%) was found to be similar in both groups. Both DM and HTN were found among 16.7% of the participants in Group A and 0.0% in Group B. Combined DM, HTN, and IHD were not found in any group of participants.

The level of pro-inflammatory and anti-inflammatory cytokines among study participants is presented in Table 2. The mean IL-2 level (in pg/mL) was observed to be 0.56±1.75 (SD) for patients in Group A, 0.0±0.0 (SD) for patients in Group B, and 0.0±0.0 (SD) for healthy controls, with a p-value of 0.141, which was not statistically significant. However, significantly higher levels of IL-6 and IFN-γ (in pg/mL) were observed among Group A patients, with the means detected as 216.95±147.78 (SD) and 0.98±0.95 (SD) in Group A and 94.29±128.79 (SD) and 0.41±0.61 (SD) in Group B patients. Additionally, TNF-α was detectable at 0.86±1.81 and 0.25±1.37 in Group A and Group B patients, respectively, and in controls, it was 0.0±0.0 (p-value=0.444). The level of anti-inflammatory cytokines (IL-10) was found to be higher in Group A patients than in the other two groups, which is statistically significant. The mean IL-10 level (in pg/mL) was observed to be 17.92±21.87 for Group A patients, 5.38±6.73 for Group B patients, and 1.62±1.65 for healthy individuals.

**Table 2:** The level of pro-inflammatory and anti-inflammatory cytokines among study participants Data are presented as mean ± SD. ^α^A significant mean difference between Group A and Group B patients. ^β^A significant mean difference between Group A and Group C. *p-value was determined by a one-way ANOVA test.

Cytokines	Group A (n=30)	Group B (n=30)	Group C (n=10)	p-Value*
Pro-inflammatory markers				
IL-2 (pg/mL)	0.56±1.75	0.0±0.0	0.0±0.0	0.141
IL-6 (pg/mL)	216.95±147.78	94.29±128.79^α^	1.08±1.97^β^	<0.001
TNF-α (pg/mL)	0.86±1.81	0.25±1.37	0.0±0.0	0.444
IFN-γ (pg/mL)	0.98±0.95	0.41±0.61^α^	0.15±0.28^β^	0.002
Anti-inflammatory marker				
IL-10 (pg/mL)	17.92±21.87	5.38±6.73^α^	1.62±1.65^β^	0.002

The mean values (pg/mL) of IL-2 for the age groups 41-50, 51-60, and 61-70 were 0.0, 0.82, and 0.90 for Group A, and for Group B, it was found to be 0.0 for all age groups. The mean values (pg/mL) of IL-6 for the age groups 41-50, 51-60, and 61-70 are 184.93, 243.04, and 316.55 for Group A and 74.66, 98.98, and 125.69 for Group B, respectively. The mean value (pg/mL) of TNF-α for the 41-50, 51-60, and 61-70 age groups is 0.0, 0.45, and 1.27 for Group A and 0.0, 0.0, and 0.54 for Group B. The mean level of IFN-γ (pg/mL) in the age groups 41-50, 51-60, and 61-70 is 0.74, 1.04, and 2.01 in Group A, and in Group B, it is 0.15, 0.41, and 0.66, respectively. The mean IL-10 (pg/mL) for the age groups 41-50, 51-60, and 61-70 are 16.72, 16.97, and 18.78 in Group A and 4.62, 4.68, and 7.37 in Group B, respectively. Both Group A and Group B showed a positive relationship between the mean level of cytokines (pg/mL) and the age group in years. IL-2, IL-6, TNF-α, IFN-γ, and IL-10 were higher in Group A (severe group) than in Group B (non-severe group), but were found to be significant only for IFN-γ in the age group 51-60 and for IL-10 in the age group 61-70.

The concentration and comparison of cytokines in terms of gender among the participants of Group A and Group B are shown in Table 3. The mean concentration (pg/mL) of IL-2, IL-6, TNF-α, IFN-γ, and IL-10 for Group A male participants were 0.62±0.33, 197.82±144.44, 1.18±3.59, 1.07±1.02, and 18.26±24.06, respectively, and for Group A female participants, it was 0.55±0.29, 269.56±153.51, 0.0±0.0, 0.76±0.74, and 16.98±15.63, respectively. For participants in Group B, the mean concentration (pg/mL) of IL-2, IL-6, TNF-α, IFN-γ, and IL-10 was 0.0±0.0, 89.19±112.92, 0.49±1.93, 0.37±0.54, and 3.89±3.14 in men, respectively, and for women, it was 0.0±0.0, 99.38±146.79, 0.0±0.0, 0.44±0.68, and 6.88±8.90, respectively.

**Table 3:** Comparison of cytokine levels in terms of gender among the participants of Group A and Group B *Independent Student's t-test was done. Data are expressed as mean ± SD.

Grouping of patients	Cytokines	Male	Female	p-Value*
Group A	IL-2 (pg/mL)	0.62±0.33	0.55±0.29	0.843
	IL-6 (pg/mL)	197.82±144.44	269.56±153.51	0.246
	TNF-α (pg/mL)	1.18±3.59	0.0±0.0	0.370
	IFN-γ (pg/mL)	1.07±1.02	0.76±0.74	0.443
	IL-10 (pg/mL)	18.26±24.06	16.98±15.63	0.891
Group B	IL-2 (pg/mL)	0.0±0.0	0.0±0.0	1.00
	IL-6 (pg/mL)	89.19±112.92	99.38±146.79	0.833
	TNF-α (pg/mL)	0.49±1.93	0.0±0.0	0.142
	IFN-γ (pg/mL)	0.37±0.54	0.44±0.68	0.778
	IL-10 (pg/mL)	3.89±3.14	6.88±8.90	0.230

Table 4 shows the mean concentration (pg/mL) of IL-6, IFN-γ, and IL-10 among patients with the most common comorbidities (DM, HTN, and COPD). The mean IL-6, IFN-γ, and IL-10 among the Group A DM patients were 187.34±155.03, 0.82±0.93, and 22.56±28.37, respectively, and for Group B, it was 184.27±147.74, 0.0±0.0, and 3.89±2.56, respectively. No significant differences were observed among the patients with DM in Group A and Group B. HTN showed significantly higher concentrations of IL-6 (201.47±130.24 in Group A and 57.59±45.06 in Group B). IFN-γ (1.00±0.94 in Group A and 0.41±0.64 in Group B) and IL-10 (15.19±45.06 in Group A and 4.67±4.43 in Group B) were non-significant. The mean values of IL-6, IFN-γ, and IL-10 in the patients in Group A with COPD were 253.61±163.46, 21.58±27.69, and 0.60±0.74, respectively. No COPD patient was present in Group B. Among the patients who had both DM and HTN from Group A, the mean IL-6, IFN-γ, and TNF-α were 229.46±105.46, 1.21±0.75, and 27.08±15.15, respectively. No participant was found to have combined DM, HTN, and COPD in both groups.

**Table 4:** The level of the cytokine among patients having comorbidities in both groups (Group A and Group B) Data are expressed as mean ± SD. *p-value was determined by independent Student's t-test. ^∞^p-value could not be calculated as at least one group was empty. DM: diabetes mellitus; HTN: hypertension; COPD: chronic obstructive pulmonary disease.

Comorbidities	Cytokines	Group A	Group B	p-Value*
DM (n=6) (A-4, B-2)	IL-6 (pg/mL)	187.34±155.03	184.27±147.74	0.323
	IFN-γ (pg/mL)	0.82±0.93	0.0±0.0	0.427
	IL-10 (pg/mL)	22.56±28.37	3.89±2.56	0.588
HTN (n=13) (A-8, B-5)	IL-6 (pg/mL)	201.47±130.24	57.59±45.06	0.030
	IFN-γ (pg/mL)	1.00±0.94	0.41±0.64	0.217
	IL-10 (pg/mL)	15.19±45.06	4.67±4.43	0.349
COPD (n=7) (A-7, B-0)	IL-6 (pg/mL)	253.61±163.46	-	∞
	IFN-γ (pg/mL)	0.60±0.74	-	∞
	IL-10 (pg/mL)	21.58±27.69	-	∞
DM+HTN (n=5) (A-5, B-0)	IL-6 (pg/mL)	229.46±105.46	-	∞
	IFN-γ (pg/mL)	1.21±0.75	-	∞
	IL-10 (pg/mL)	27.08±15.15	-	∞

## Discussion

COVID-19 has emerged as a global health challenge. The clinical spectrum of the SARS-CoV-2 infection is broad, ranging from asymptomatic cases to severe pneumonia and fatalities [11]. Hospitalized patients often face pneumonia, with about 30% developing sustained lung injury. The prevailing belief is that elevated cytokine levels contribute to destructive capillary bed damage, tissue edema, fluid accumulation in the lungs, and a robust inflammatory cell infiltrate, ultimately leading to acute respiratory distress syndrome (ARDS) [12]. Early prediction of COVID-19 severity proves challenging clinically, necessitating biochemical markers. This study focuses on evaluating the association of inflammatory and anti-inflammatory cytokines with COVID-19 severity.

A total of 60 RT-PCR-positive COVID-19 patients and 10 apparently healthy individuals were included in the study. The demographic data of the study participants revealed interesting patterns. The three groups (Group A, Group B, and Group C) differed significantly in terms of age distribution. Group A had an older population, with a mean age of 60.73 years, compared to Group B (57.13 years) and Group C (48.10 years). However, older people are more susceptible to COVID-19 infection, which may be due to their low-functioning immune systems that fail to keep the immune response to certain pathogens in check and the presence of other comorbidities like DM, HTN, COPD, and HF [13]. Additionally, there were variations in gender distribution, with Group A having more males (73.3%) than Group B (50.0%) and Group C (60.0%). The male predominance may be due to their higher chance of exposure to occupational activities, and another important factor is the smoking habit, which is more common in males than females and causes symptomatic COVID-19 disease due to their compromised lungs [14].

Most participants in all groups were from urban areas during COVID-19 episodes, with Group A having 56.7%, Group B having 53.3%, and Group C not specified. As this study was conducted in a single center from Dhaka city, most of the participants were from urban areas; however, urban people were affected more during the peak of the pandemic due to high population density, mass movements of the population at railway stations, bus stops, markets, etc. Socioeconomic status, based on monthly income, classified participants into low-, middle-, and high-income groups. Interestingly, most participants in all groups were from middle-income families, indicating a potential socioeconomic factor in the study. The presentation of complaints during COVID-19 episodes showed that fever was universal among patients in both groups. Cough was prevalent in both severe (Group A) and non-severe (Group B) patients, but certain symptoms such as anosmia, respiratory distress, sore throat, and runny nose varied significantly between the two groups. Group A had higher incidences of respiratory distress, sore throat, and runny nose, and these differences were statistically significant. The comorbidities among the groups showed varying prevalence rates, with Group A having higher percentages for DM, HTN, COPD, and HF compared to Group B. Interestingly, the percentages of ischemic heart disease (IHD) were similar in both groups. Combined, DM, HTN, and IHD were not present in any group. Similarly to previous studies, the presence of comorbidities was observed to have a positive correlation with disease severity.

Analysis of pro-inflammatory and anti-inflammatory cytokine levels revealed noteworthy findings. Although IL-2 levels were not significantly different between groups, IL-6 and IFN-γ levels were higher in Group A, indicating a potentially more pronounced immune response in severe cases. However, IL-2 was reported to play a significant role in the severity of COVID-19 disease by enhancing NK cell cytolytic activity in different studies [15,16]. Regarding IL-6 and IFN-γ, similar findings have been reported from previous studies in COVID-19 patients [17]. IL-6, produced in response to tissue damage and infections and during certain viral infections, may promote virus survival and/or exacerbation of clinical disease [18]. Thus, excessive IL-6 levels in COVID-19 patients correlate with severe disease, including pulmonary inflammation, lung damage, and multiple organ failure. IFN-γ, the primary activator of macrophages and neutrophils and produced by NK cells, T lymphocytes, and other cells of the immune system, has also been reported to play an important role in innate and adaptive immunity [19]. TNF-α, which regulates the inflammatory processes of infectious diseases, had its levels elevated in patients with COVID-19 and were higher in severe disease, resulting in a poor prognosis [5]. However, in this study, TNF-α was detectable at very low levels in both groups. The anti-inflammatory cytokine IL-10 showed a significant increase in Group A compared to Group B and healthy controls. The early induction of IL-10 upon COVID-19 infection during the innate immunity phase in the lung likely serves as a countermeasure to inflammation caused by other pro-inflammatory cytokines. As the levels of endogenous IL-10 increase, it functions as an immune-activating/pro-inflammatory agent to stimulate the production of members of the cytokine storm [6]. IL-10 directly expands cytotoxic effector CD8+ T cells; hyper-activation of adaptive immunity in COVID-19 patients greatly exacerbates the disease condition [20]. Therefore, IL-10 inhibits the activity of T cells, NK cells, and macrophages during acute infection, which, although necessary to eradicate viruses, is also a key factor in causing tissue damage [20]. Thus, IL-10 can reduce the damage to collateral tissues while preventing the successful elimination of viruses [10]. Several studies have shown that levels of IL-10 result in poor disease progression in patients with COVID-19 [21]. Higher concentrations of IL-2, IL-6, IL-8, and TNF-α were also reported in ICU patients with COVID-19 in comparison to non-ICU patients [22]. The drastic initial rise of IL-10 in COVID-19 severe patients is a very much distinguishing and seemingly paradoxical observation in contrast to its classical anti-inflammatory role [23].

Further analysis demonstrated a positive relationship between cytokine levels and age, with certain cytokines showing higher concentrations in Group A (the severe group) compared to Group B (the non-severe group). Several studies showed that IL-6, IFN-γ, and IL-10 levels increased with age. In Brazil, approximately 51% of COVID-19 occurs in older individuals (over 60 years of age), and it was observed that older people had a higher chance of disease severity and were considered one of the risk groups for COVID-19 [24]. A higher level of IL-6 was observed among female severe COVID-19 patients in this study, though it was not statistically significant. The exact cause of the high level of IL-6 in female participants is not clear. However, it may be due to the small female sample size of study participants. The concentration of cytokines among patients with comorbidities (DM, HTN, and COPD) varied, with HTN patients showing significantly higher levels of IL-6 in both groups. A similar result was also reported from a study in China, where almost 27% of COVID-19 patients had hypertension, developed acute respiratory distress syndrome (ARDS), and died [25].

In summary, this study highlighted age-related differences in cytokine levels, the impact of comorbidities on cytokine concentrations, and significant variations in symptoms and comorbidity prevalence between severe and non-severe COVID-19 cases. These findings can inform future research and contribute to a better understanding of the immune response in different demographic and health conditions during COVID-19.

There were certain challenges and limitations to this study. CBA is a very expensive method, and it was not possible to increase the sample size of the current study. As individual kits were not available during the study period, a human Th1/Th2/Th17 CBA kit was chosen. Hence, this study was carried out with a limited sample size due to financial and time constraints. Moreover, the influence of the patients' medical history was not included in this study. Thus, further study with a larger sample size and advanced statistical analysis like multivariate analysis is recommended to evaluate these findings.

## Conclusions

COVID-19 challenges modern medical science; however, to satisfy the urgency of devising a new approach to therapy, a clear understanding of the disease's immunopathogenesis is imperative. Despite the limitations, the present study was able to shed some light on the association of cytokines, especially IL-6, IL-10, and IFN-γ, with COVID-19 disease severity. Taking this into consideration, this study might help to consolidate the assessment of the mentioned cytokines as a predictive tool for COVID-19 disease progression.
